# Self‐Adaptive Biomimetic Nanocomposite Hydrogel Orchestrates Dual‐Targeting and On‐Demand H_2_S Gasotransmission for Multifactorial Diabetic Wound Therapy

**DOI:** 10.1002/advs.78108

**Published:** 2026-10-06

**Authors:** Kefan Wu, Xiaoxue Yang, Yonghang Liu, Yi Xiao, Shiyu Meng, Ao Li, Yi Zhun Zhu, Hui Guo, Xiaolin Wang

**Affiliations:** ^1^ School of Pharmacy and State Key Laboratory of Mechanism and Quality of Chinese Medicine Macau University of Science and Technology Taipa Macao China

**Keywords:** diabetic wound healing, hydrogen sulfide, microenvironment‐adaptive, nanocomposite hydrogel, neutrophil‐mimetic, targeted delivery

## Abstract

Diabetic wounds remain a persistent clinical challenge due to the intertwined pathologies of chronic inflammation, oxidative stress, bacterial colonization, and impaired neovascularization. Conventional hydrogel dressings, however, typically lack both the spatiotemporal precision and synergistic multifunctionality that are required to counteract the complex pathological drivers of diabetic wound chronicity. Here, we present a biomimetic nanocomposite hydrogel (QN‐S‐HG) that integrates neutrophil‐membrane‐coated quercetin‐loaded nanoparticles with a pH‐responsive hydrogen sulfide (H_2_S) releasing hyaluronic acid network. The neutrophil‐mimetic nanoparticles achieve dual‐targeted delivery to inflammatory cells and bacteria, simultaneously exerting anti‐inflammatory, antioxidant, and broad‐spectrum antibacterial effects. Concurrently, the H_2_S donor S‐propargylcysteine (SPRC) is covalently conjugated via dynamic Schiff base bonds to the hydrogel scaffold, enabling acid‐triggered release in the pathological wound milieu to stimulate angiogenesis. Remarkably, the crosslinker‐free and hydrogen‐bond‐mediated hyaluronic acid network endows the hydrogel with injectability, self‐healing, and tissue‐adhesive properties, ensuring conformal coverage and sustained local bioactivity. In a diabetic rat full‐thickness wound model, QN‐S‐HG markedly accelerates closure, promotes macrophage repolarization, enhances neovascularization, and facilitates orderly collagen remodeling with hair follicle regeneration. By orchestrating targeted pharmacotherapy, microenvironment‐triggered gaseous signaling, and adaptive material mechanics, this platform addresses the multifaceted pathophysiology of diabetic wounds and offers a clinically translatable paradigm for advanced regenerative wound care.

## Graphical abstract

A self‐adaptive and biomimetic nanocomposite hydrogel is engineered to orchestrate dual‐targeting and on‐demand H_2_S gasotransmission for multifactorial diabetic wound therapy. By seamlessly integrating antibacterial, antioxidant, anti‐inflammatory, immunoregulatory, and pro‐angiogenic functions within a single platform, this hydrogel dynamically remodels the pathological wound microenvironment and effectively accelerates diabetic tissue regeneration.

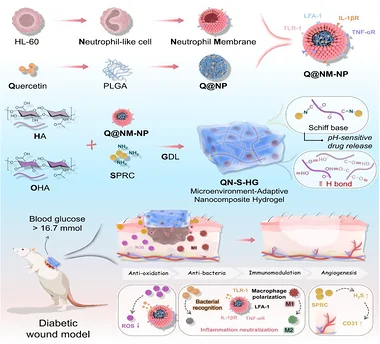

## 1 Introduction

Wound healing is a dynamic and highly coordinated process involving hemostasis, inflammation, proliferation, and tissue remodeling, with each stage orchestrated by the interplay of diverse cells and molecular factors [1, 2, 3]. In diabetic patients, however, chronic hyperglycemia not only fuels the excessive generation of reactive oxygen species (ROS) and advanced glycation end‐products, but also induces immune cell dysfunction, characterized by impaired neutrophil chemotaxis, defective macrophage efferocytosis, and a sustained bias toward the pro‐inflammatory M1 phenotype [4, 5]. These derangements converge to create a hostile wound microenvironment marked by unabated inflammation, elevated oxidative stress, and recurrent bacterial colonization [6, 7, 8]. Meanwhile, diabetes‐associated vascular insufficiency exacerbates local hypoxia and nutrient deprivation, further entrenching the wound in a non‐healing state and exposing patients to limb‐threatening complications [9, 10, 11]. As diabetes prevalence escalates globally, the development of therapeutic strategies that concurrently resolve inflammation, eradicate infection, and reinstate vascular function has become an urgent clinical priority [12, 13, 14].

Hydrogels have garnered considerable interest in wound repair owing to their high‐water content, extracellular matrix‐mimetic architecture, capacity to maintain moisture, absorb exudate, and serve as depots for bioactive cargo [15, 16, 17, 18]. Recent advances have yielded multifunctional systems integrating antibacterial, antioxidant, adhesive, and self‐healing properties [19, 20, 21, 22]. As typical examples, theranostic hydrogels [23], injectable antibacterial conductive hydrogels [24], engineered probiotic‐loaded hydrogels [25], and vaso‐neural interoperable hydrogels [26] have demonstrated robust wound healing efficacy. Yet, most existing hydrogel dressings operate as passive delivery vehicles, lacking the ability to sense and dynamically adapt to the evolving pathological wound milieu [27]. Moreover, their inherent hydrophilicity often compromises the loading of hydrophobic therapeutics, while uncontrolled burst release limits sustained efficacy [28]. Consequently, existing hydrogel dressings primarily function as passive drug reservoirs, lacking the ability to actively sense and respond to pathological microenvironmental cues [29]. To overcome these challenges, it is imperative to develop multifunctional hydrogel dressings that actively engage with the wound microenvironment through targeted delivery and on‐demand cargo release [30, 31].

Cell‐membrane‐coating nanotechnology offers a compelling route to bestow synthetic carriers with native biological functions, including pathogen recognition, inflammatory tropism, and cytokine neutralization [32]. Neutrophil membranes, in particular, display a repertoire of adhesion receptors (LFA‐1), pattern‐recognition receptors (TLR‐1/2), and inflammatory cytokine receptors (TNFR, IL‐1βR), equipping nanoparticles with dual targeting to activated endothelium and bacteria, while simultaneously serving as decoys for pro‐inflammatory mediators [33]. Concurrently, gasotransmitter‐based therapy, especially hydrogen sulfide (H_2_S), has emerged as a potent pro‐angiogenic and immunomodulatory strategy in tissue repair [34]. Among various H_2_S donors, *S*‐propargyl‐cysteine (SPRC) possesses a distinct H_2_S‐generating mechanism. Unlike conventional exogenous donors that primarily release H_2_S through direct dissolution, hydrolysis, or chemical decomposition, SPRC promotes endogenous H_2_S production by enhancing the activity and expression of cystathionine‐γ‐lyase (CSE), thereby enabling a more physiologically regulated and sustained elevation of H_2_S [35]. This CSE‐dependent mechanism has shown therapeutic potential in inflammatory and regenerative disorders, and SPRC has also demonstrated beneficial effects in skin wound repair [36]. In addition to its biological advantages, the primary amino group of SPRC provides intrinsic chemical compatibility with aldehyde‐functionalized biomaterials. However, achieving localized and on‐demand H_2_S delivery remains challenging, as systemic administration suffers from rapid clearance and off‐target effects [37]. Therefore, we hypothesize that integrating neutrophil‐membrane‐mimetic targeting with acid‐labile H_2_S donation into a hydrogel matrix can actively remodel the pathological diabetic wound microenvironment.

Herein, we engineer a microenvironment‐adaptive nanocomposite hydrogel (QN‐S‐HG) that synergistically combines neutrophil‐mimetic dual targeting with pH‐labile H_2_S release to rectify the diabetic wound milieu. Quercetin (Q), a natural flavonoid with hypoglycemic, antioxidant, anti‐inflammatory, and antibacterial activities [38], is encapsulated within neutrophil‐membrane‐coated nanoparticles (Q@NM‐NP) to ensure targeted delivery to inflamed and infected sites [39, 40]. Moreover, the hydrogel scaffold, composed of hyaluronic acid and its oxidized derivative, is physically crosslinked solely by hydrogen bonds, obviating chemical crosslinkers and conferring injectability, self‐healing, and strong tissue adhesion, different from conventional HA hydrogels [41]. Crucially, the endogenous H_2_S donor *S*‐propargylcysteine (SPRC) is covalently grafted to the network via Schiff base bonds, enabling pH‐triggered cleavage and localized H_2_S elevation in the acidic wound bed to drive neovascularization [10, 42, 43]. Through this integrative design, QN‐S‐HG orchestrates multi‐stage therapeutic actions, including dual‐targeted drug delivery, microenvironment‐responsive H_2_S generation, and adaptive material properties to rebalance inflammation, eliminate infection, and reinstate vascular regeneration. In a diabetic rat model, the system achieves accelerated closure, enhanced epithelial maturation, robust collagen deposition, and hair follicle neogenesis, underscoring its potential as a clinically translatable strategy for complex diabetic wound management (Figure 1).

**FIGURE 1:**
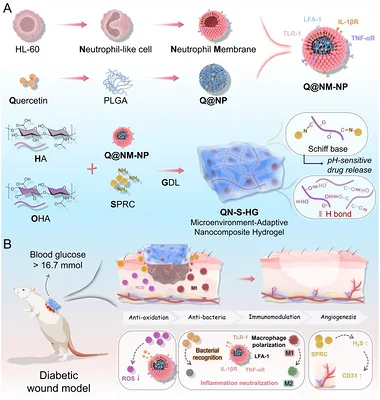
Design and therapeutic mechanism of the biomimetic nanocomposite hydrogel (QN‐S‐HG) for diabetic wound healing. (A) Schematic diagram depicting the synthesis of the QN‐S‐HG hydrogel. (B) The multifunctional QN‐S‐HG hydrogel promotes diabetic wound repair by orchestrating a cascade of biological events, including alleviating oxidative stress, combating bacterial infection, neutralizing inflammation, inducing macrophage repolarization toward the anti‐inflammatory M2 phenotype, and stimulating angiogenesis.

## 2 Results and Discussions

### 2.1 Preparation and Characterization of Q@NM‐NP

In the first place, neutrophil membrane‐coated and Q‐loaded nanoparticles (Q@NM‐NP) were readily fabricated (Figure 2A). More specifically, the nanocore Q@NP was successfully prepared using the single emulsion‐solvent evaporation method. Concurrently, neutrophil membranes (NM) were isolated from DMSO‐differentiated HL‐60 cells. The biomimetic product, Q@NM‐NP, was then fabricated by fusing the Q@NP core with the NM shell via sonication. Successful membrane camouflage was confirmed through a combination of morphological and physicochemical characterizations. Transmission electron microscopy (TEM) revealed spherical Q@NM‐NP with a uniform core‐shell structure, displaying a diameter of ∼200 nm with the outer membrane shell measured to be ∼10 nm in thickness (Figure 2B). Dynamic light scattering (DLS) analysis corroborated this core‐shell architecture, showing a slight but distinct increase in hydrodynamic diameter from 185 ± 3.36 nm for the uncoated Q@NP to 200 ± 4.55 nm after membrane coating (Figure 2C). Furthermore, the zeta potential of Q@NM‐NP (−23 ± 3.5 mV) was intermediate between that of the bare Q@NP core and the isolated neutrophil membrane, confirming the alteration of the nanoparticle surface by the membrane coating (Figure 2D). Finally, the formulation exhibited high drug loading efficiency, with an encapsulation efficiency (EE) of 84.9% ± 2% and a drug loading capacity (DLC) of approximately 12.3% ± 0.6% (Figure S1), establishing its potential for effective therapeutic delivery.

**FIGURE 2:**
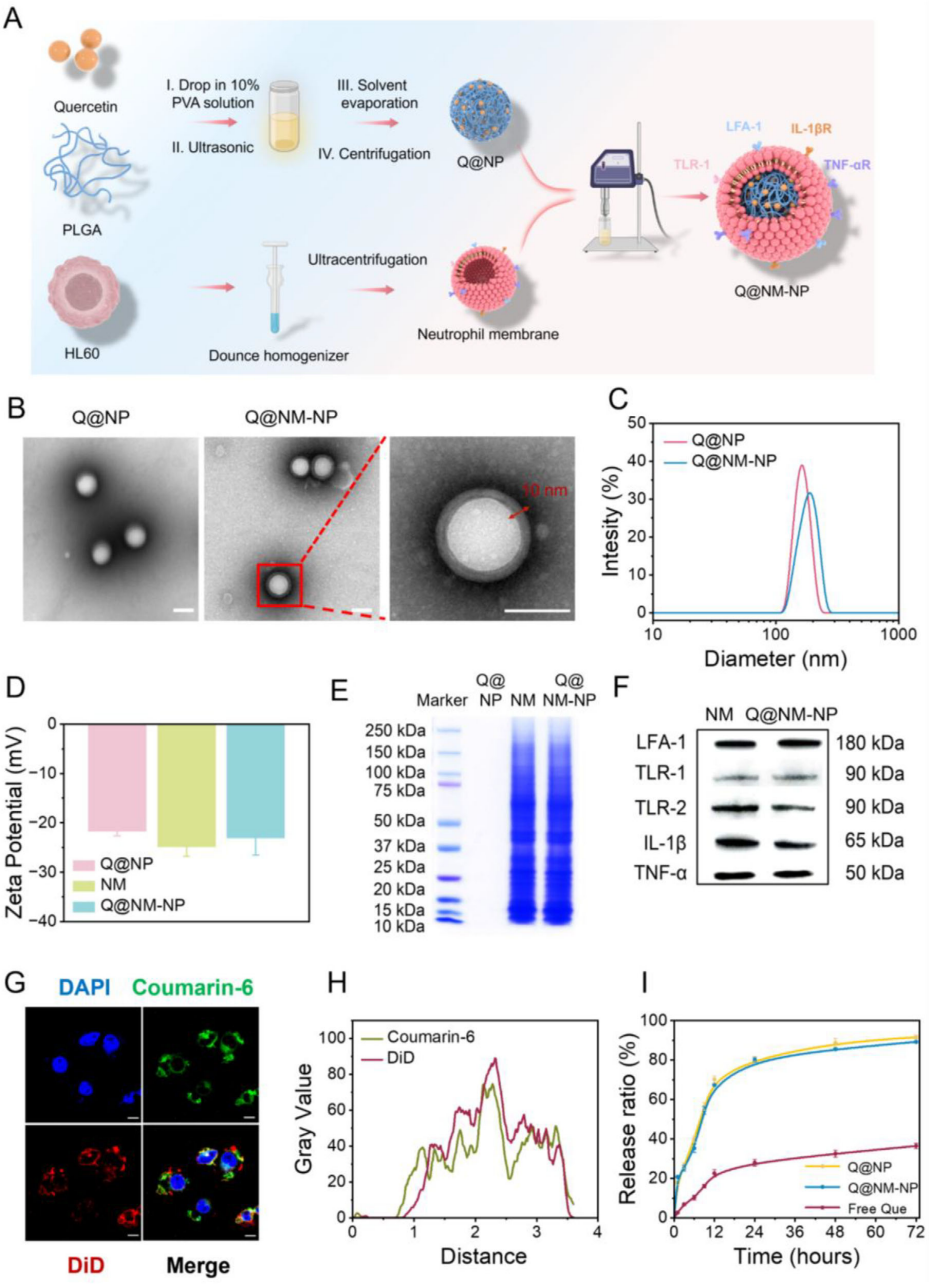
Preparation and characterization of neutrophil membrane‐coated biomimetic nanoparticle Q@NM‐NP. (A) Schematic illustration of the fabrication process of Q@NM‐NP. (B) Morphology of Q@NP and Q@NM‐NP by TEM. Scale bar = 150 nm. (C) Hydrodynamic diameters of Q@NP and Q@NM‐NP. (D) Zeta potentials of Q@NP, NM, and Q@NM‐NP. Protein composition of Q@NP, NM, and Q@NM‐NP detected by (E) Coomassie blue staining and (F) Western blotting analysis. Colocalization analysis of Coumarin‐6‐labeled NP and DiD‐labeled NM by (G) CLSM (blue, DAPI; green, Coumarin‐6; red, DiD) and (H) ImageJ. Scale bar = 10 µm. (I) The cumulative Q release kinetics from Q@NP and Q@ NM‐NP.

The successful coating of the neutrophil membrane onto the nanocore was further confirmed by SDS‐PAGE analysis. As expected, Q@NM‐NP retained the full protein profile characteristic of natural neutrophil membranes, whereas bare Q@NP showed no detectable protein bands (Figure 2E). Western blot analysis further identified the presence of key functional receptors on Q@NM‐NP, including the adhesion receptor lymphocyte function‐associated antigen 1 (LFA‐1), the pathogen recognition receptors Toll‐like receptor 1 and 2 (TLR‐1/2), and the inflammatory cytokine receptors tumor necrosis factor‐alpha (TNF‐α) receptor (TNFR) and interleukin‐1 beta (IL‐1β) receptor (IL‐1βR) (Figure 2F). This preserved receptor repertoire suggested that the biomimetic nanoparticles could achieve dual targeting to inflamed tissues and bacteria, while exerting complementary anti‐inflammatory effects through neutralization of inflammatory mediators.

To evaluate the cellular uptake and intracellular localization of Q@NM‐NPs, fluorescence‐labeled nanoparticles were co‐incubated with TNF‐α‐stimulated human umbilical vein endothelial cells (HUVECs). Confocal laser scanning microscopy (CLSM) revealed strong co‐localization of the green (Coumarin‐6‐labeled PLGA core) and red (DiD‐labeled membrane) signals, confirming that the nanoparticles maintained their structural integrity during the interaction process (Figure 2G,H). This selective adhesion was attributed to the specific interaction between LFA‐1 on the nanoparticle surface and its ligand intercellular adhesion molecule 1 (ICAM‐1), which is widely known to be upregulated on activated HUVECs (Figure S2).

We next evaluated whether this targeting capability translated into enhanced cellular uptake in inflammatory cells. Using lipopolysaccharide (LPS)‐stimulated RAW 264.7 macrophages to mimic the pro‐inflammatory microenvironment of diabetic wounds, we found that NM‐NP exhibited markedly enhanced uptake by inflamed macrophages compared to non‐stimulated controls (Figure S3). Notably, the neutrophil membrane coating also reduced non‐specific uptake by resting macrophages, suggesting a dual role in both enhancing targeted delivery to inflamed sites and minimizing off‐target clearance.

Given the presence of inflammatory cytokine receptors on the nanoparticle surface, we hypothesized that Q@NM‐NP could also function as nanosponges to neutralize pro‐inflammatory mediators. To verify this point, we incubated increasing concentrations of NM‐NP with fixed amounts of TNF‐α and IL‐1β. Both cytokines were adsorbed in a concentration‐dependent manner (Figure S4). Fitting the binding data to the Hill equation yielded half‐maximal binding (IC_50_) values of 122.1 µg mL^−^^1^ for TNF‐α and 85.6 µg mL^−^^1^ for IL‐1β, demonstrating potent inflammatory cytokine‐neutralizing activity.

Finally, we examined whether the membrane coating affected the drug release profile. As shown in Figure 2I, both Q@NP and Q@NM‐NP exhibited similar sustained release kinetics, indicating that the coating process did not compromise the drug encapsulation or release behavior. In contrast, free Q showed minimal release due to its poor aqueous solubility, underscoring the necessity of nanoparticle encapsulation for effective drug delivery. Collectively, these results demonstrate that Q@NM‐NP successfully inherits the multifunctional properties of natural neutrophils, including inflamed endothelium targeting, macrophage uptake, cytokine neutralization, and sustained drug release, positioning it as a promising biomimetic platform for diabetic wound therapy.

### 2.2 Fabrication and Characterization of QN‐S‐HG

Building upon the successful fabrication of the nanoparticles, we subsequently developed a class of hyaluronic acid (HA)‐based multifunctional hydrogels (HG) by hydrogen bond‐mediated physical crosslinks. In the first place, HA polymer with ultra‐high molecular weight (1.5 × 10^6^ g/mol) was gently oxidized to yield aldehyde‐functionalized HA (OHA), where the chemical structure is verified in Figure S5. Then, both HA and OHA were homogeneously dissolved in alkaline media to form a highly concentrated polymer solution. Under such circumstances, the solubilities of both polymers were upregulated since the intramolecular hydrogen bonds were dramatically weakened with the deprotonation of carboxyl and hydroxy groups [44]. Subsequently, glucono‐*delta*‐lactone (GDL) solution was added dropwise into the alkaline solution to adjust the pH. Upon the gradual neutralization of the system with the autohydrolysis of GDL, the negatively charged hydroxyl groups were reverted to neutral, generating abundant intramolecular hydrogen bonds and triggering the formation of the hydrogel [45]. To promote the hydrogel performance in wound healing, SPRC was covalently grafted into the network via Schiff‐base bonds, which endows the SPRC with pH‐sensitive cleavage capability to adjust the pathological condition. Eventually, Q@NM‐NP was integrated into the system to form a nano‐composite hydrogel (QN‐S‐HG).

The chemical composition of the hydrogel was further verified by a Fourier transform infrared spectrometer (FTIR) (Figure 3A). The characteristic absorption peak of ─C═O at 1728 cm^−1^ is observed in the S‐HG and QN‐S‐HG hydrogels, indicating that the Schiff base reaction is achieved between OHA and SPRC. More importantly, compared with the precursor polymer, the ^1^H NMR spectrum of S‐HG exhibited a newly emerged weak resonance in the characteristic imine proton region at approximately δ 8.3 ppm, which can be assigned to the ─CH═N─ moiety generated through the condensation of aldehyde and amino groups (Figure S6). These complementary results provide further evidence for the formation of dynamic Schiff‐base linkages between OHA and SPRC.

**FIGURE 3:**
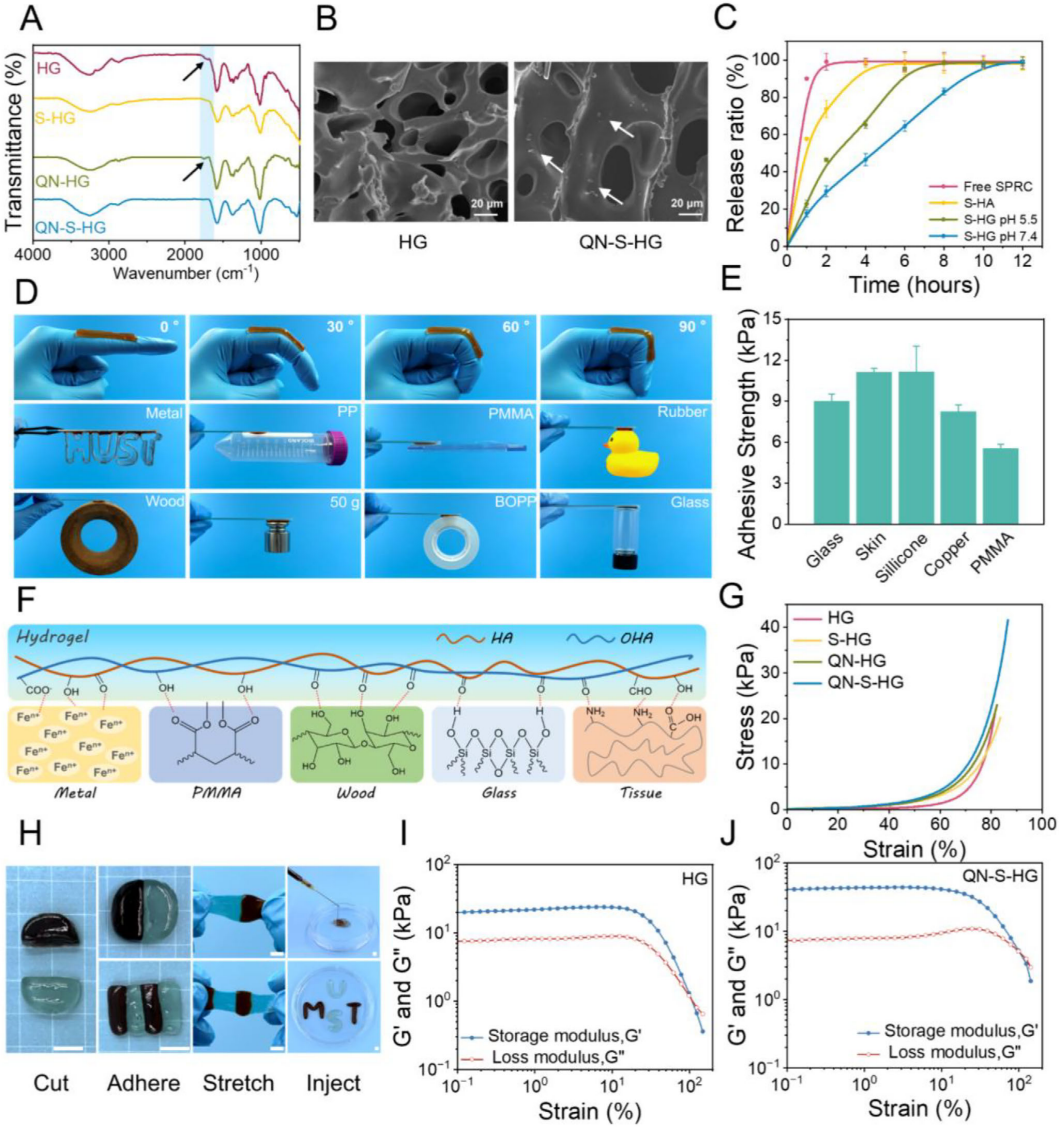
Characterizations of nano‐composite hydrogels. (A) Fourier transform infrared spectra of hydrogels at the wavenumber of 4000–600 cm^−1^. (B) Morphology of HG and QN‐S‐HG hydrogels observed by SEM. Scale bar = 20 µm. (C) The SPRC release kinetics from QN‐S‐HG hydrogel in different media at 37°C. S‐HA represents SPRC incorporated into the HG hydrogel via physical blending. (D) Representative images of QN‐S‐HG adhering to gloves with fingers bending at four degrees (0°, 30°, 60°, and 90°), and the photos of hydrogels adhering to different substrates. (E) Adhesive strength of the QN‐S‐HG hydrogel on different substrates with a lap‐shear method. (F) Schematic illustration of the adhesion mechanism. (G) Typical uniaxial compression stress‐strain curves for HG, S‐HG, QN‐HG, and QN‐S‐HG hydrogels. Deformation rate = 100 min.min^−1^. (H) Self‐healing and injectable behaviors of HG and QN‐S‐HG hydrogels. Scale bar = 10 mm. Viscoelastic performances of (I) HG and (J) QN‐S‐HG hydrogels. Frequency = 1 Hz, Temp. = 25°C.

In addition, the scanning electron microscope (SEM) showed that the nanoparticles were evenly dispersed in the pores of the hydrogel, which indicated the successful construction of a nano‐composite system (Figure 3B).

Then, the drug release behaviors of the hydrogel were systematically investigated with SPRC and Q, which demonstrate multiple biological effects, including angiogenesis, antibacterial, and antioxidant activities [42, 46]. As shown in Figure 3C, the SPRC agent exhibits a significantly enhanced release rate in a weakly acidic environment. Since the SPRC is grafted onto the hydrogel by Schiff‐base bonds, the dynamic bonds endow the hydrogel with high sensitivity to the pathological low‐pH wound microenvironment, enabling it to release the active agent and accelerate the diabetic wound healing process. The loading efficiency of S‐HG was presented in Figure S7. Moreover, the release of Q from the hydrogels was monitored in pH 7.4 PBS at 37°C. Complete drug release from the hydrogel matrix extended over 4 days, ensuring potential therapeutic effects for the treatment period while reducing the frequency of dressing changes for chronic diabetic wound management (Figure S8).

To further determine whether SPRC release translated into actual H_2_S generation at the cellular level, H_2_S production was monitored in inflammatory HUVECs over 48 h. As shown in Figure S9, the intracellular H_2_S concentrations in the HG and QN‐HG groups remained close to the basal level throughout the observation period. In contrast, both S‐HG and QN‐S‐HG induced a time‐dependent elevation of H_2_S, reaching maximal levels at approximately 12 h, followed by a gradual decline toward the basal level at 48 h. This temporal profile indicates that incorporation of SPRC into the hydrogel provides a sustained rather than transient elevation of cellular H_2_S. Consistently, WSP‐1 fluorescence imaging revealed markedly enhanced intracellular green fluorescence in the SPRC‐containing S‐HG and QN‐S‐HG groups, whereas only weak fluorescence was detected in the Control, HG, and QN‐HG groups (Figure S10). Collectively, these results directly confirm that the SPRC‐functionalized hydrogel effectively elevates intracellular H_2_S levels, thereby providing experimental support for the proposed H_2_S‐mediated therapeutic mechanism.

Given the critical role of adhesion in wound dressing performance, we characterized the adhesive behavior of QN‐S‐HG under physiologically relevant conditions (Figure 3D). Experimental results demonstrated its high adhesion to a diverse range of substrate materials, including metals, wood, glass, and rubber [47]. As shown in Figure 3E, quantitative analysis with a conventionally applied lap‐shear test revealed high adhesion strengths across different material interfaces, with the highest adhesive force of 11 kPa, satisfying the essential requirements for diabetic wound dressing employment. The adhesion mechanism can probably be attributed to multiple non‐covalent interactions, particularly hydrogen bonding and ionic interactions between the carboxyl/hydroxyl groups of the hydrogel and the polar substrates (Figure 3F) [47, 48]. In addition, QN‐S‐HG exhibits favorable mechanical properties to withstand the dynamic stresses of wound movement and compression during the healing process. Uniaxial compression testing was conducted to assess the mechanical behavior of the hydrogel, yielding the strain‐stress curves presented in Figure 3G. Comparative analysis indicated that the QN‐S‐HG hydrogel exhibited a two‐fold enhancement in mechanical properties relative to the blank HG hydrogel. This substantial improvement can be ascribed to the nanoparticle encapsulation effect, which effectively fills the porous network of the base hydrogel matrix, thereby reinforcing its structural integrity and load‐bearing capacity [49].

To better meet the requirements of wound repair, we evaluated the self‐healing properties of the QN‐S‐HG hydrogel (Figure 3H). To this end, samples were sectioned into geometrically distinct fragments and placed in proximate contact on the experimental substrate. Remarkably, within a 3‐min incubation period, both hydrogel variants demonstrated complete structural reintegration, with the QN‐S‐HG composite exhibiting particularly enhanced tensile characteristics post‐healing. The multiple non‐covalent interactions, especially the hydrogen bonds between carboxyl and hydroxyl units, contribute to this profile.

Owing to the dynamic 3D structure, the QN‐S‐HG hydrogel displayed satisfactory injectability, a critical feature for clinical applications requiring conformation to complex wound topographies. This rheological property ensures comprehensive wound coverage through precise material deposition. Viscoelastic characterization via strain sweep analysis (0.1%–1% strain range) confirmed that both HG and QN‐S‐HG hydrogels maintained their structural integrity in the linear viscoelastic regime (Figure 3I,J), with storage modulus (G') values consistently exceeding loss modulus (G'') values (1.99 and 4.1 kPa for HG and QN‐S‐HG, respectively). Progressive strain advancement induced a characteristic gel‐to‐sol transition, marked by the crossover of G′′ over G′, thereby rendering the hydrogel highly amenable to smooth syringe‐based injection. The rapid gelation behavior was further verified by time‐dependent rheological analysis. Upon addition of GDL, both G′ and G″ increased rapidly as the polymer network developed, and a crossover between G′ and G″ was observed at approximately 91 s (Figure S11). Thereafter, G′ remained higher than G″ and continuously increased, indicating the rapid transition from a predominantly viscous precursor solution to a stable elastic hydrogel network. Such a short gelation time is advantageous for injectable wound dressing applications by allowing rapid in situ formation after administration.

### 2.3 In Vitro Analysis of Biocompatibility and Antibacterial Ability

The biocompatibility of the biomimetic nanoparticle Q@NM‐NP and its composite hydrogel formulation QN‐S‐HG was systematically evaluated with cell viability and hemolysis tests [50, 51, 52, 53]. To this end, NIH3T3 fibroblasts and HUVECs were exposed to varying concentrations of Q@NM‐NP (10–1000 µg mL^−^^1^) for 24 h, after which cellular metabolic activity was assessed using the Alamar Blue assay (Figure S12) [54]. Notably, both cell lines maintained >90% viability across the entire concentration range, demonstrating the excellent cytocompatibility of Q@NM‐NP and supporting its suitability for further in vivo evaluation. Cytotoxicity of the hydrogel formulations was similarly assessed by measuring NIH3T3 and HUVEC viability after 24 h of co‐incubation (Figure 4A,B). All four hydrogel groups demonstrated high cell viability approaching 100%, indicating that neither SPRC incorporation nor nanocomposite integration compromised cellular safety. Hemocompatibility was evaluated via hemolysis assays using a 2% red blood cell (RBC) suspension incubated with the nanoparticles or hydrogels for 3 h (Figure S13). Deionized water and PBS (pH 7.4) served as positive and negative controls, yielding hemolysis rates of 100% and 0%, respectively. Q@NM‐NP induced minimal hemolysis (<5%) across the entire concentration range (10–1000 µg mL^−^^1^). Likewise, all hydrogel formulations consistently exhibited hemolysis rates below the 5% threshold, confirming excellent blood compatibility (Figure 4C). Collectively, these results establish the robust biosafety profile of the QN‐S‐HG nanocomposite hydrogel, providing a solid foundation for its application in tissue repair.

**FIGURE 4:**
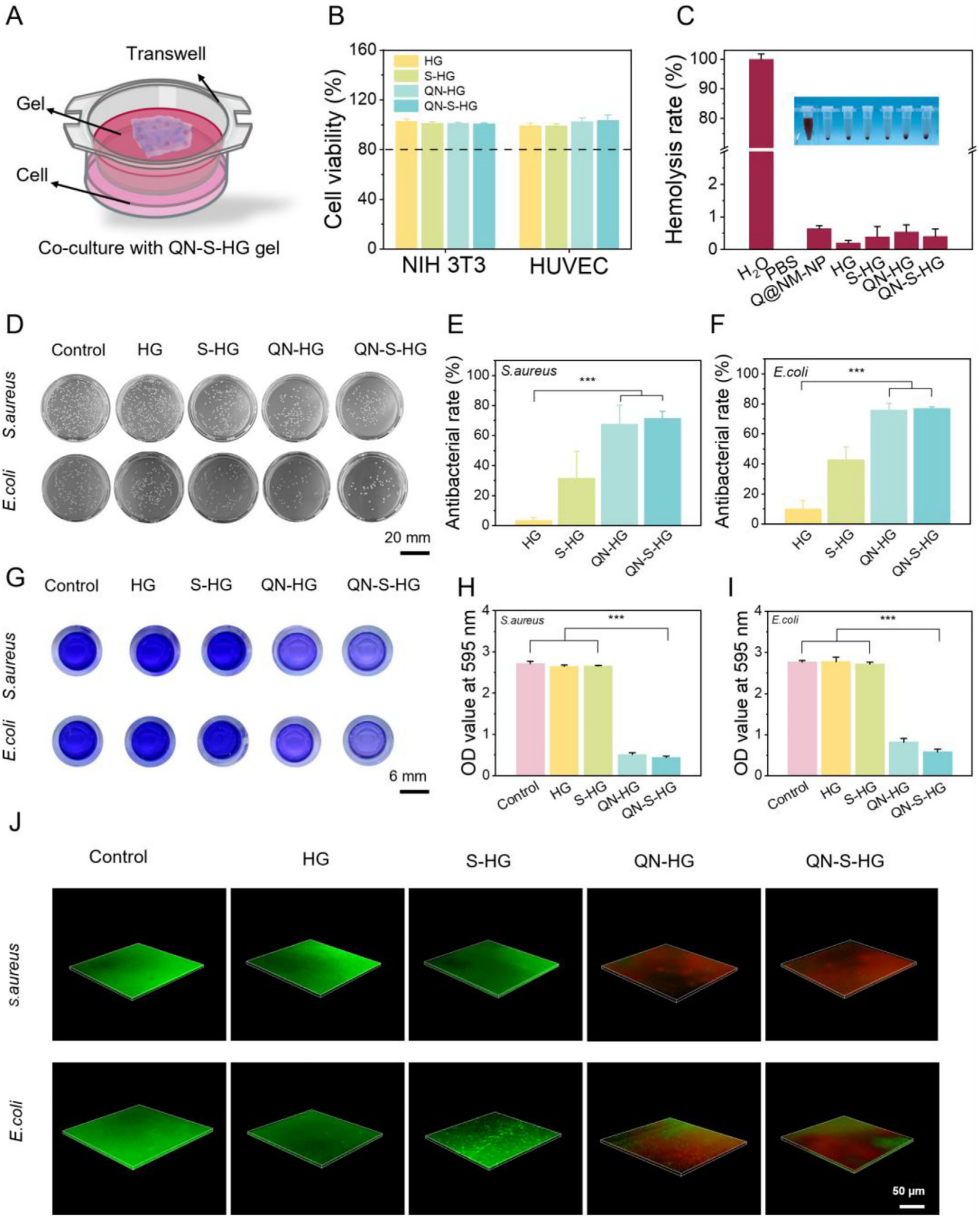
Biocompatibility and antibacterial properties of QN‐S‐HG hydrogels in vitro. (A) Illustration of the cell‐gel co‐culture model. (B) The cell viability of NIH‐3T3 and HUVECs cells co‐cultured with different hydrogels. (C) Hemolysis rates of different groups. (D) Representative images of bacterial colonies on agar plates. Scale bar = 20 mm. Corresponding bacterial viability of different gels against (E) *S. aureus* and (F) *E. coli*. (G) Representative crystal violet staining images of biofilm after various treatments. Scale bar = 6 mm. Quantitative analysis of (H) *S. aureus* and (l) *E. coli* of OD value at 595 nm. (J) 3D fluorescence images of biofilms after various treatments. Scale bar = 50 µm. Data were expressed as means ± SD, *n* = 3. Data were expressed as means ± SD, *n* = 3. Statistical significance among various groups was determined by one‐way ANOVA with Tukey's post‐hoc test (ns: non‐significant, **p < 0.01; ***p < 0.001).

Bacterial infections severely impede wound healing in chronic diabetic wounds, necessitating the development of effective antibacterial dressings. To this end, we performed a series of in vitro antibacterial assays to assess their potential [55, 56]. This study first evaluated the antibacterial activity of NP, Q@NP, and Q@NM‐NP against Gram‐positive *Staphylococcus aureus* (*S. aureus*) and Gram‐negative *Escherichia coli* (*E. coli*) using the plate counting method based on the broad‐spectrum antimicrobial activity of Q [40]. As a result, both Q@NP and Q@NM‐NP significantly reduced bacterial colonies compared to the control and blank nanoparticle groups (Figure S14). Notably, Q@NM‐NP outperformed Q@NP, achieving a substantially higher inhibition rates of 77.68% ± 3.6% and 68.32% ± 2.48% against *S. aureus* and *E. coli* strains. This enhanced bactericidal effect is likely attributable to improved pathogen recognition and binding mediated by surface Toll‐like receptor membrane proteins (TLR‐1/2) [57].

The antibacterial activity of the nanocomposite hydrogel QN‐S‐HG was further assessed by co‐culturing hydrogel pieces with *S. aureus* or *E. coli* in fresh LB broth for 6 h [58, 59], followed by optical density measurement at 600 nm (OD600) and enumerating colony‐forming units (CFUs). The results demonstrated that the blank HG (control) exhibited minimal antibacterial activity against both *S. aureus* and *E. coli* (Figure 4D–F). In contrast, the S‐HG hydrogel exerted a moderate bacteriostatic effect by promoting H_2_S generation, which disrupts bacterial antioxidant enzyme activity, triggers the superoxide response regulatory system, and induces oxidative stress. Notably, the nanocomposite hydrogels displayed significantly enhanced antibacterial performance, with QN‐S‐HG exhibiting the highest efficacy, achieving inhibition rates of 71.34% ± 4.88% against *S. aureus* and 76.77% ± 1.26% against *E. coli*. These findings underscore the superior antibacterial properties of QN‐S‐HG, highlighting its potential to mitigate wound infection risks in biomedical applications.

Given that persistent biofilm formation represents a major obstacle to the treatment of chronic wound infections, we further investigated the antibiofilm activity of the hydrogel formulations against *S. aureus* and *E. coli*. Crystal violet staining revealed dense biofilm formation in the Control and HG groups, whereas substantially reduced staining was observed after treatment with QN‐HG and QN‐S‐HG (Figure 4G). Quantitative analysis of the residual biofilm biomass at 570 nm further confirmed that both QN‐HG and QN‐S‐HG markedly reduced biofilm formation compared with the Control, HG, and S‐HG groups for both bacterial strains (Figure 4H,I). Consistently, 3D CLSM imaging of bacterial biofilms demonstrated dense viable bacterial populations in the untreated and HG groups, whereas the QN‐S‐HG group markedly disrupted the biofilm architecture and increased bacterial death (Figure 4J). Collectively, these results demonstrate that QN‐S‐HG exhibits pronounced antibiofilm activity in addition to its antibacterial effect against bacteria, further supporting its potential for the management of biofilm‐associated chronic wound infections.

### 2.4 In Vitro Assessment of Cell Migration, Macrophage Polarization, Antioxidant, and Angiogenesis

The migratory capacity of cells plays a pivotal role in wound healing [60]. In this study, epithelial cells (HaCaT) and HUVEC were selected to assess the potential of QN‐S‐HG hydrogel in promoting re‐epithelialization and angiogenesis (Figure S15) [61, 62]. To simulate the inflammatory microenvironment characteristic of diabetic wounds, lipopolysaccharide (LPS) was employed to stimulate two distinct types of tissue cells [63]. A standardized scratch assay was then performed to establish an in vitro wound model. An inverted microscope was utilized to quantitatively assess dynamic changes in re‐epithelialization at predetermined intervals (0, 12, 24, and 36 h). The LPS‐stimulated group served as the positive control (Control (+)), while the unstimulated group was the negative control (Control (−)).

As illustrated in Figure 5A, scratch assay results revealed significant impairment in cell migration capacity in the Control (+) group compared to the Control (‐) group. Notably, the LPS‐stimulated group maintained a substantial scratch area even at the 36 h time point, demonstrating the inhibitory effects of inflammation on wound closure. In contrast, all hydrogel‐treated groups exhibited markedly enhanced migratory activity, with scratch areas progressively approximating those observed in the normal Control (‐) group. Figure 5B presents the quantitative analysis of wound closure rates. After 12 h of incubation, the blank HG hydrogel showed modest pro‐migratory effects, achieving only 30.1% ± 5.8% wound closure. In comparison, drug‐loaded hydrogel formulations (SHG, QN‐HG, and QN‐S‐HG) demonstrated significantly enhanced efficacy, with closure rates of 37.7% ± 6.8%, 40.7% ± 4.0%, and 41.8% ± 5.8%, respectively. Notably, after 36 h of treatment, the QN‐S‐HG group exhibited a migration rate nearly equivalent to that of the Control (−) group, suggesting this composite material possesses superior potential for facilitating wound re‐epithelialization in diabetic conditions (Figure 5C).

**FIGURE 5:**
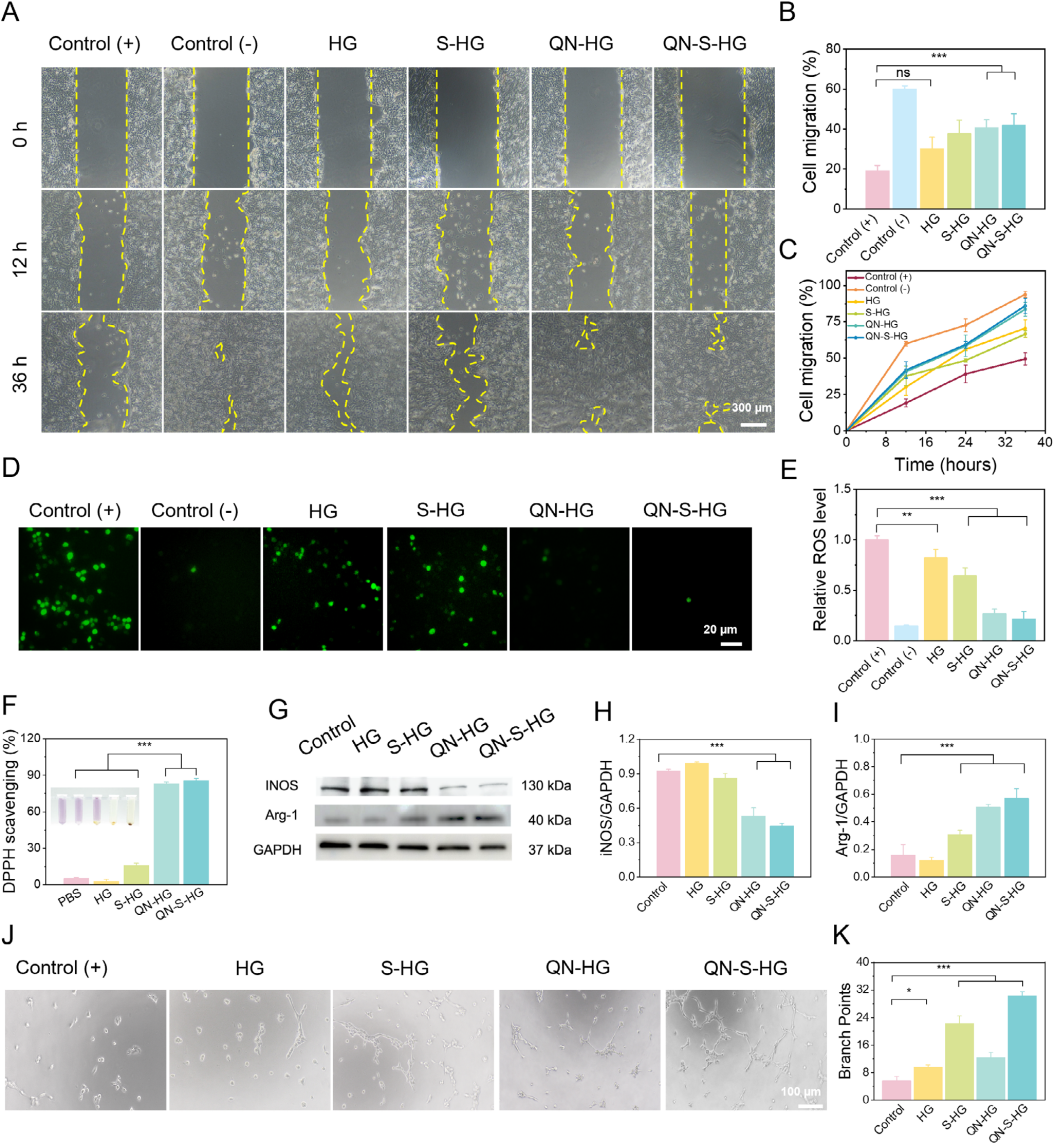
Therapeutic effects of QN‐S‐HG in vitro. (A) Representative images of HaCat cell migration treated with HG, S‐HG, QN‐HG, and QN‐S‐HG. (B) Quantitative statistics of HaCat migration after 12 h and (C) all time intervals. Scale bar = 300 µm. Control (+) group: LPS stimuli; Control (−) group: No LPS stimuli. (D) Fluorescent microscope images of intracellular ROS production as probed by DCFH‐DA (green) and (E) quantitative analysis of relative ROS levels after different treatments. Control (+) group: LPS stimuli; Control (−) group: No LPS stimuli. Scale bar = 20 µm. (F) The optical image and quantification of the DPPH scavenging efficiency of various groups. (G) Expression levels of iNOS and Arg‐1 were measured by Western blot analysis. Quantification of (H) iNOS and (I) Arg‐1 expression by ImageJ. (J, K) Representative images of the tube‐forming and statistical analysis of branch points at 6 h after different treatments. Scale bar = 100 µm. Data were presented as mean ± SD for *n* = 3. Statistical significance among various groups was determined by one‐way ANOVA with Tukey's post‐hoc test (ns: non‐significant, ***p < 0.001).

Excessive production of reactive oxygen species (ROS) by immune cells such as macrophages and neutrophils can lead to metabolic disorders, cell apoptosis, and tissue damage in wounds [64]. Therefore, DCFH‐DA, a fluorescent probe widely used for intracellular ROS detection, functions through enzymatic hydrolysis by intracellular esterase and subsequent ROS‐mediated oxidation, ultimately generating quantifiable fluorescent signals [4, 65]. Lipopolysaccharide (LPS) was employed to induce oxidative stress in macrophages, simulating the pathological microenvironment of diabetic wounds, except for the negative control group (Control (−)) [66]. As demonstrated in Figure 5D, the positive control group Control (+) exhibited extensive green fluorescence after DCFH‐DA loading, indicative of substantial ROS generation. In contrast, the QN‐HG and QN‐S‐HG groups exhibited fluorescence intensities comparable to those of the Control (−) group, with only negligible green fluorescence. Notably, their fluorescence signals were significantly attenuated relative to not only the Control (+) group but also the blank hydrogel (HG) and hydrogen sulfide donor‐only (S‐HG) groups (Figure 5E). These results unequivocally demonstrate the exceptional ROS‐scavenging capacity of QN‐S‐HG, which can be primarily attributed to the Q encapsulated within the nanocomposites.

Additionally, the antioxidant capacities of different nanoparticles (Figure S16) and composite hydrogels were evaluated using the DPPH scavenging assay. As illustrated in Figure 5F, the QN‐HG and QN‐S‐HG hydrogels demonstrated robust antioxidant performance, achieving DPPH scavenging rates of 83.3% ± 1.34% and 85.8% ± 1.4%, respectively. By contrast, the S‐HG group exhibited a negligible DPPH clearance of only 15.8% ± 1.72%. These findings unambiguously confirm that the superior antioxidant activity of the QN‐S‐HG hydrogel is primarily derived from the Q moiety. These results unequivocally demonstrate the exceptional ROS‐scavenging capacity of QN‐S‐HG, which can be primarily attributed to the Q encapsulated within the nanocomposites.

To assess macrophage polarization, RAW 264.7 cells were stimulated with LPS and subsequently treated with hydrogels of varying compositions [67, 68]. Subsequently, western blot analysis was conducted using inducible nitric oxide synthase (iNOS) and arginase‐1 (Arg‐1) as canonical biomarkers for M1‐type (pro‐inflammatory) and M2‐type (anti‐inflammatory) macrophages, respectively (Figure 5G) [69]. As illustrated in Figure 5H,I, the Control, HG, and S‐HG groups exhibited elevated iNOS expression but minimal Arg‐1 expression, suggesting a predominant M1 phenotypic state. In contrast, the QN‐HG and QN‐S‐HG groups demonstrated a significant reversal in this expression profile, with marked suppression of iNOS and upregulation of Arg‐1. Notably, quantitative analysis revealed that in the QN‐S‐HG group, the normalized expression levels of iNOS and Arg‐1 relative to GAPDH were 44.6% ± 2.1% and 57.1% ± 7.1%, respectively, indicating a substantial shift toward the M2 phenotype. These findings strongly suggest that QN‐S‐HG effectively induces macrophage repolarization from the pro‐inflammatory M1 to the anti‐inflammatory M2 phenotype, thereby modulating the inflammatory microenvironment and potentially enhancing diabetic wound healing.

Vascular regeneration is a rate‐limiting step in diabetic wound repair. To assess the angiogenic potential of the nanocomposite hydrogel QN‐S‐HG, an endothelial tube formation assay was performed [42]. As illustrated in Figure 5J,K, treatment with the hydrogen sulfide donor‐loaded hydrogel (S‐HG) markedly enhanced capillary‐like network formation relative to the control group. Intriguingly, while Q alone demonstrated modest pro‐angiogenic activity, the QN‐S‐HG formulation exhibited a significantly greater number of branch points compared to the S‐HG group. These findings strongly suggested that Q and SPRC exerted potent synergistic effects, collectively amplifying the proliferative, migratory, and morphogenic capacities of HUVECs to drive robust tube formation.

Collectively, the in vitro results confirm that this nanocomposite hydrogel integrates multiple therapeutic functions, including antibacterial activity, oxidative stress alleviation, inflammation neutralization, immunomodulation, and pro‐angiogenic effects, positioning it as a promising candidate for diabetic wound treatment.

### 2.5 Evaluation of Diabetic Wound Healing In Vivo

The wound healing potential of QN‐S‐HG was evaluated by a full‐thickness wound model in the Type I diabetic model [70]. A standardized circular full‐thickness excisional wound model (9 mm in diameter) was created on the dorsal skin of rats [71]. Wound repair was assessed across different treatment groups, as outlined in Figure 6A. The untreated control group served as a baseline to evaluate the wound‐healing efficacy of various hydrogel formulations. Type I diabetes was induced in all rats through intraperitoneal administration of streptozotocin (STZ). Following 14 days of blood glucose monitoring, all STZ‐treated rats exhibited significantly elevated blood glucose levels (24.0–33.3 mmol/L), far exceeding the normal physiological range (Figure S17).

**FIGURE 6:**
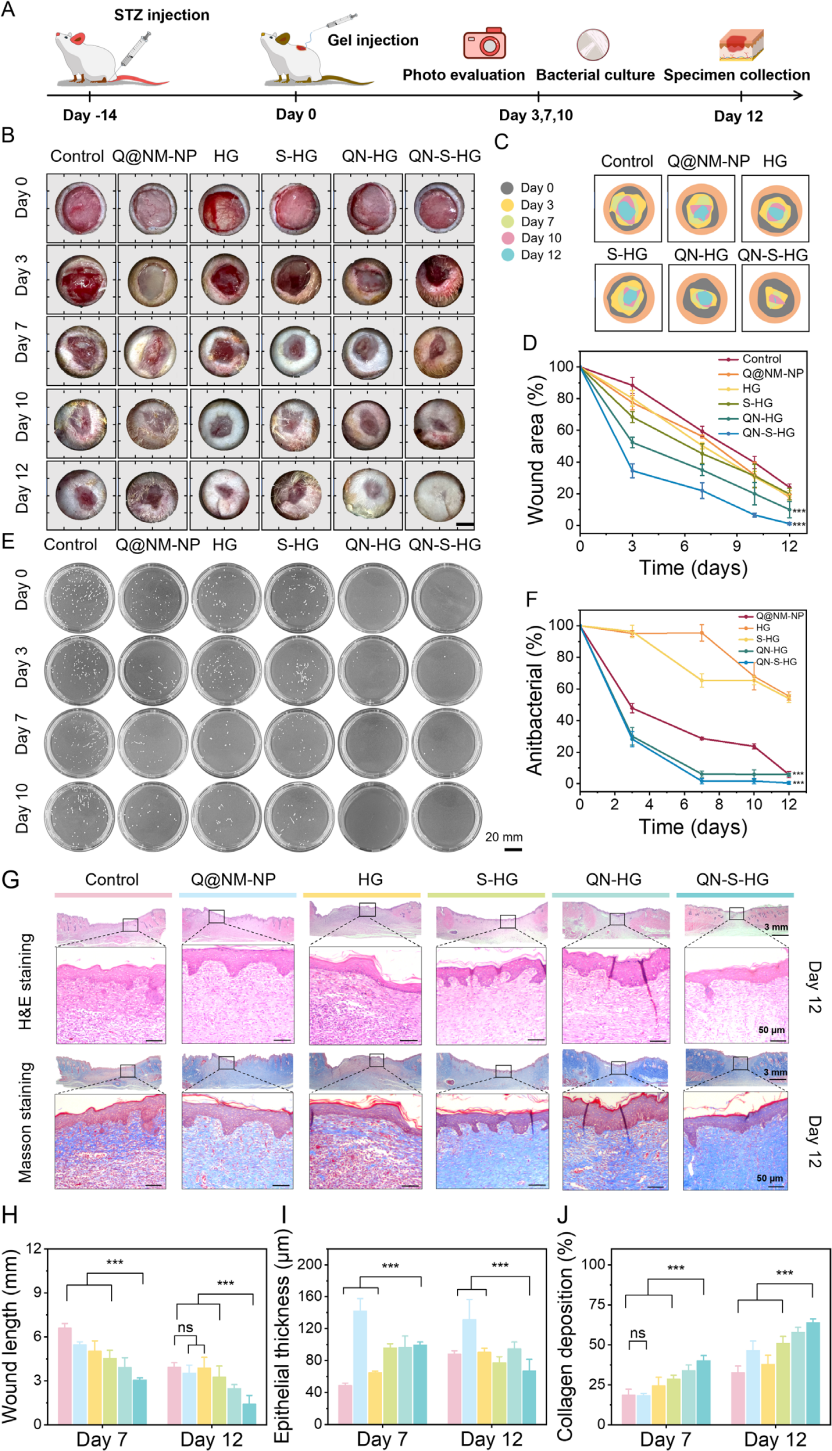
Diabetic wound healing by QN‐S‐HG. (A) Schematic diagram of QN‐S‐HG hydrogel for diabetic wound repair. (B) Representative image of wounds on days 0, 3, 7, 10, and 12. Control group: untreated wounds. Scale bar = 4 mm. (C) Wound healing process of different treatment groups. (D) Statistical analysis of the wound area during the treatment process (*n* = 6). (E) Representative images of bacterial growth on agar plates at the wound site. Scale bar = 20 mm. (F) Quantitative statistics of bacterial infection rates in the wounds of different groups. (G) Representative images of H&E and Masson's trichrome (MT) staining of wound tissues on day 12. Corresponding quantification of (H) wound length, (I) Epithelial thickness, and (J) Collagen deposition. Scale bar = 50 µm. Data were expressed as means ± SD, *n* = 6. Statistical significance among various groups was determined by one‐way ANOVA with Tukey's post‐hoc test (ns: non‐significant, **p < 0.01; ***p < 0.001).

Figure 6B presents representative wound images across experimental groups from day 0 to 12 post‐treatment. To facilitate quantitative analysis of wound healing progression, Figure 6C illustrates the temporal changes in wound area, as measured by ImageJ software. Strikingly, the nanocomposite hydrogel group (QN‐S‐HG) demonstrated significantly enhanced wound closure as early as day 3, with markedly reduced wound areas compared to both the untreated control (Control) and blank hydrogel (HG) groups, highlighting its rapid therapeutic onset. In contrast, the Q@NM‐NP group exhibited suboptimal efficacy, limited therapeutic efficacy due to rapid drug clearance after administration, which failed to maintain effective drug concentration at the wound site. Furthermore, Figure 6D depicts the healing kinetics through comparative wound closure curves for each treatment group. By the endpoint of the study (day 12), QN‐S‐HG‐treated wounds achieved near‐complete closure, with residual wound area measuring only 1.13% ± 0.7% of the initial size, alongside visible hair follicle regeneration as a key indicator of functional skin recovery. In comparison, the control group retained 24% ± 2.1% of the original wound area. Intermediate therapeutic outcomes were observed in the Q@NM‐NP (17.9% ± 1.7%), HG (18.4% ± 3.3%), S‐HG (19.8% ± 3.7%), and QN‐HG groups, all of which outperformed the control but remained inferior to QN‐S‐HG. These findings underscore the superior reparative capacity of QN‐S‐HG, which synergistically combines sustained drug release with bioactive scaffold properties to accelerate diabetic wound healing. The differential efficacy among treatment modalities further validates the critical role of formulation design in achieving optimal therapeutic outcomes.

Secondary bacterial infections severely exacerbate diabetic wound healing by instigating a self‐perpetuating inflammatory cycle. The resulting immune‐hostile microenvironment thwarts fibroblast activity, angiogenesis, and extracellular matrix remodeling, thereby trapping the wound in a persistent, non‐healing state. In this study, bacterial burden in diabetic wounds across experimental groups was quantitatively assessed by plate counting on days 3, 7, 10, and 12. As illustrated in Figure 6E, the bacterial infection kinetics revealed distinct patterns among treatment groups. Both QN‐S‐HG and QN‐HG demonstrated comparable antimicrobial efficacy, achieving remarkably low bacterial survival rates of 0.5% ± 0.9% and 0.6% ± 1%, respectively, by day 12 post‐treatment (Figure 6F). Meanwhile, the Q@NM‐NP group also exhibited a rapid reduction in bacterial infection rate to 5.8% ± 2.1%. However, the S‐HG group and the HG group showed unsatisfactory antibacterial effects, with a bacterial infection rate of 19.8% ± 0.1% on day 12. These findings substantiate that QN‐S‐HG effectively mitigates wound bacterial colonization, with quercetin serving as the primary antimicrobial agent. The hydrogel matrix contributes additional protective benefits through its physical barrier function, thereby synergistically enhancing the overall antibacterial performance.

To evaluate the therapeutic efficacy of QN‐S‐HG in diabetic wound healing, wound tissues were collected on days 7 (Figure S18) and 12 for histological examination (Figure 6G). Wound closure was first assessed by measuring the wound width from H&E‐stained sections. On day 12, the QN‐S‐HG group exhibited a markedly reduced wound width (1.4 ± 0.6 mm) compared to the control group (3.9 ± 0.3 mm), as well as to the Q@NM‐NP (3.5 ± 0.6 mm), HG (3.9 ± 0.7 mm), and S‐HG (3.2 ± 0.8 mm) groups, indicating superior wound contraction and tissue regeneration (Figure 6H). Re‐epithelialization, a critical hallmark of functional wound healing, was further evaluated by measuring epidermal thickness. On day 7, the QN‐S‐HG group displayed a thicker epidermis (99 ± 4 µm) than all other groups except Q@NM‐NP (Figure 6I), suggesting an accelerated initiation of epithelial proliferation. By day 12, however, the epidermal thickness in the QN‐S‐HG group had decreased and was lower than that in all other groups. This reduction reflects the transition from a proliferative to a remodeling phase, where the initially hyperplastic epidermis matures into a stratified and well‐differentiated architecture, which isa key indicator of advanced tissue regeneration. Collectively, these histological findings demonstrate that QN‐S‐HG not only accelerates wound closure but also promotes orderly epidermal maturation.

Masson's trichrome staining was further performed to evaluate collagen deposition at the wound site, as collagen serves as the fundamental extracellular matrix component supporting tissue regeneration. By day 12, wounds treated with QN‐S‐HG exhibited dense and well‐organized collagen fibers, whereas only sparse and irregular collagen deposition was observed in the HG and S‐HG groups (Figure 6G). Quantitative analysis confirmed that the collagen content in the QN‐S‐HG group was 1.3‐ to 1.9‐fold higher than that in all other treatment groups (Figure 6J), indicating that QN‐S‐HG effectively promotes extracellular matrix synthesis and tissue remodeling.

### 2.6 Anti‐Inflammatory Effects In Vivo

The chronic inflammatory response observed in diabetic wounds significantly impairs tissue regeneration while concurrently promoting skin scarring and dermal fibrosis. Consequently, accelerating the transition from the inflammatory phase to the proliferative phase is critical for effective wound healing [72]. To comprehensively assess the therapeutic efficacy of QN‐S‐HG hydrogel in modulating local inflammation in diabetic rat wounds, immunohistochemical staining was conducted on days 7 and 12 post‐treatment. This analysis quantified the expression levels of key pro‐inflammatory cytokines (TNF‐α and IL‐1β) as well as the anti‐inflammatory cytokine interleukin‐10 (IL‐10), providing insights into the hydrogel's immunomodulatory properties.

As illustrated in Figure 7A,B, both the QN‐S‐HG and QN‐HG groups demonstrated significantly reduced levels of TNF‐α and IL‐1β compared to the control group at both evaluated time points. Quantitative analysis revealed that by day 12, TNF‐α expression in the QN‐S‐HG group decreased to 7.2% ± 0.39% (Figure 7D), while IL‐1β levels dropped to 4.5 ± 0.73% (Figure 7E). Similarly, the QN‐HG group exhibited reductions to 16% ± 0.08% and 14% ± 0.43%, respectively, indicating a substantial attenuation of inflammation. Notably, the anti‐inflammatory cytokine IL‐10 displayed a distinct temporal expression pattern (Figure 7C). On day 7, the QN‐S‐HG group exhibited a significantly higher IL‐10 level compared to the control group, suggesting an early anti‐inflammatory response. However, by day 12, IL‐10 expression in the QN‐S‐HG group was markedly lower than that in the control, Q@NM‐NP, HG, and S‐HG groups (Figure 7F). These dynamic changes implied that the QN‐S‐HG‐treated wounds had already transitioned beyond the inflammatory phase by day 12, further supporting the accelerated wound repair process observed in this group.

**FIGURE 7:**
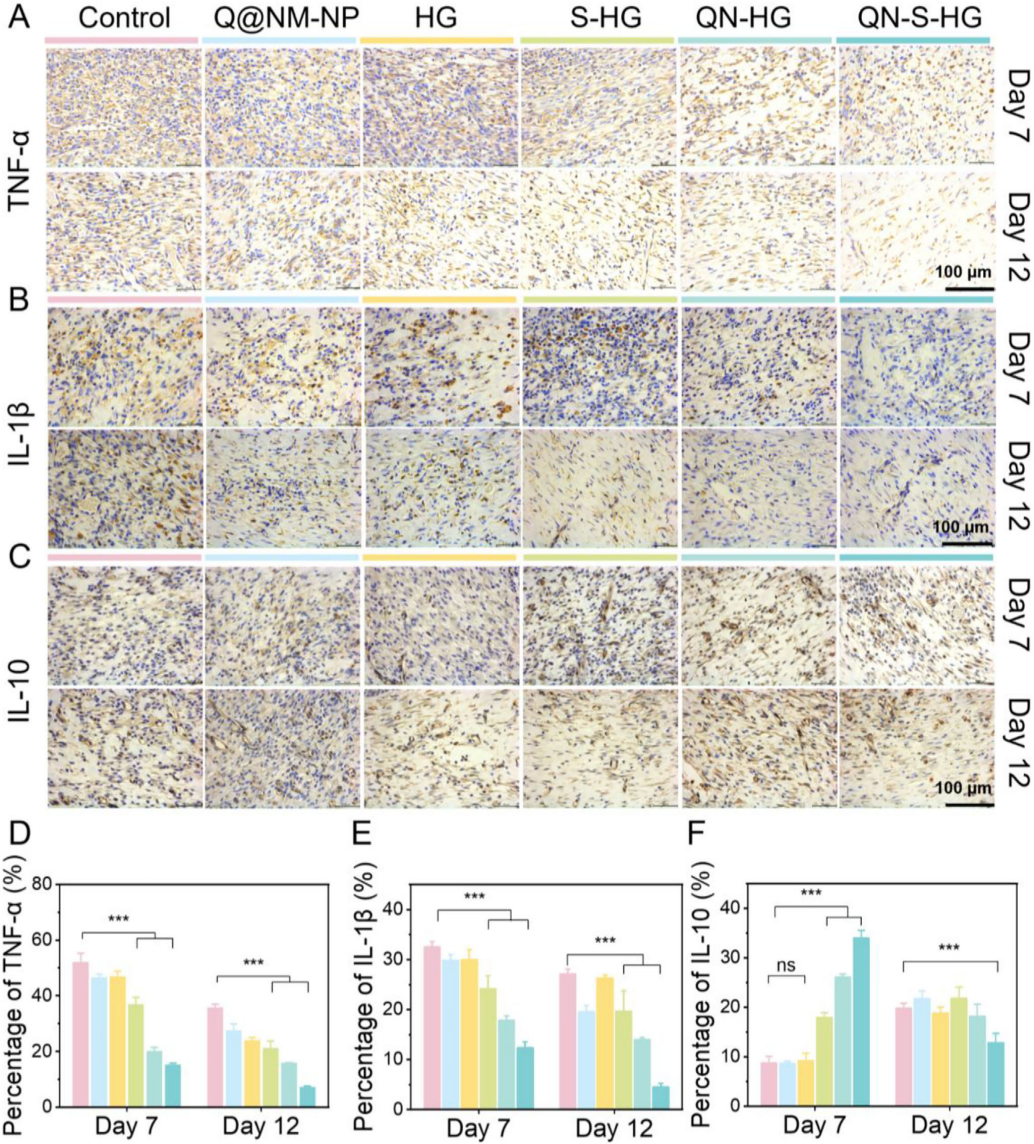
Anti‐inflammatory effects of QN‐S‐HG in vivo. Representative images of (A) TNF‐𝛼, (B) IL‐1𝛽, and (C) IL‐10 by IHC staining. Scale bar = 100 µm. Corresponding quantification of (D) TNF‐𝛼, (E) IL‐1𝛽, and (F) IL‐10 expression. Data were expressed as means ± SD, *n* = 6. Statistical significance among various groups was determined by one‐way ANOVA with Tukey's post‐hoc test (ns: non‐significant, **p < 0.01; ***p < 0.001).

### 2.7 Angiogenesis and Macrophage Repolarization In Vivo

To evaluate the angiogenic and immunomodulatory effects of different treatments in diabetic wounds, we performed CD31 immunohistochemistry to assess neovascularization and iNOS/CD206 immunofluorescence to determine macrophage phenotype switching (Figure 8A–F). Inadequate angiogenesis in diabetic wounds can result in localized ischemia and tissue necrosis, underscoring its pivotal role in wound repair and functional skin regeneration. As illustrated in Figure 8A, both the Control and blank high‐glucose (HG) groups exhibited limited neovascularization on days 7 and 12 post‐wounding. Notably, SPRC treatment significantly augmented angiogenic activity within the wound area. The S‐HG group demonstrated a marked increase in vascular density by day 7, with mature vascular structures evident (Figure 8D). Concurrently, QN‐HG also enhanced angiogenesis, albeit to a lesser extent, likely mediated by its ability to mitigate oxidative stress and suppress immuno‐inflammatory pathways in the diabetic microenvironment. Strikingly, the synergistic action of these two bioactive components in the QN‐S‐HG group yielded the most pronounced pro‐angiogenic response, as evidenced by superior neovessel formation.

**FIGURE 8:**
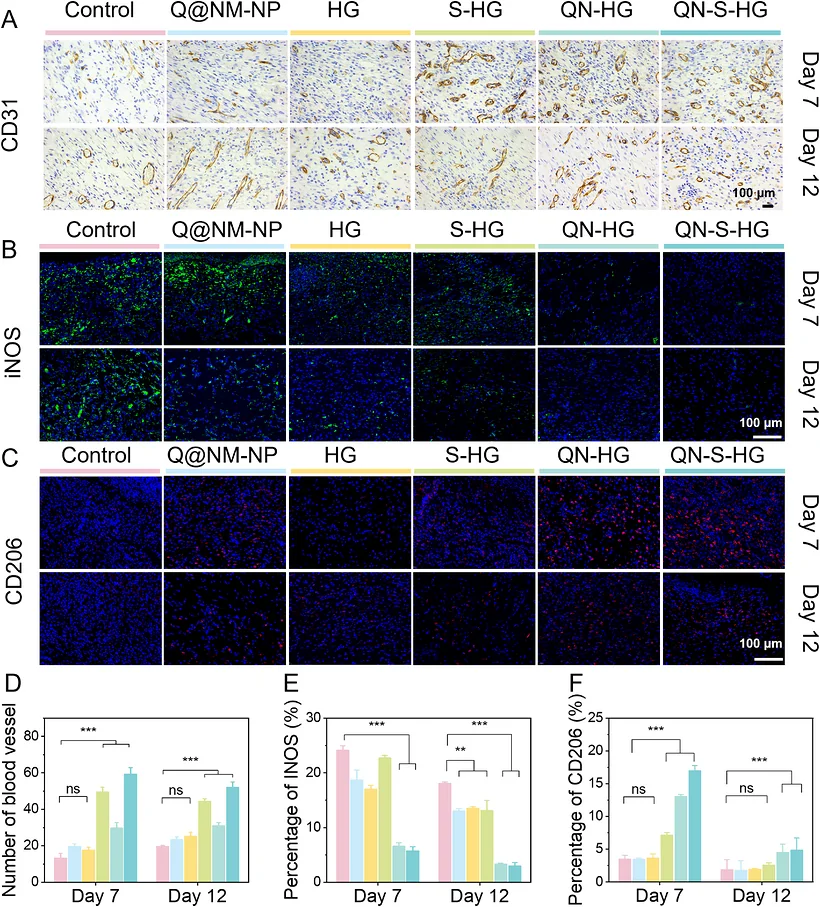
Angiogenesis and macrophage phenotype switch analysis of diabetic wounds. (A) Representative images of CD31 by IHC staining.Scale bar = 100 µm. Representative images of immunofluorescence staining of (B) iNOS (Green) and (C) CD206 (Red) on days 7 and 12. Scale bar =100 µm. (D) Corresponding quantification of CD31 expression. Quantitative analysis of (E) iNOS and (F) CD206 expression. All data are presented as mean ± SD (*n* = 6). Statistical significance among various groups was determined by one‐way ANOVA with LSD test (ns: non‐significant, **p < 0.01; ***p < 0.001).

Since the timely transition from a pro‐inflammatory (M1) to a pro‐healing (M2) macrophage phenotype governs the resolution of inflammation and the initiation of tissue regeneration, we also investigated whether the superior remodeling observed in the QN‐S‐HG cohort was driven by this immunomodulatory cascade. Immunofluorescence co‐staining for iNOS (M1) and CD206 (M2) was performed on wound sections (Figure 8B,C). QN‐S‐HG decreased iNOS‐associated and increased CD206‐associated signals in wound tissue, and directly downregulated iNOS while upregulating Arg‐1 in RAW264.7 macrophages (Figure S19), together indicating a shift toward a pro‐healing immune microenvironment. The untreated control exhibited intense iNOS immunoreactivity (18.1% ± 0.2%), indicative of a chronic, unresolved pro‐inflammatory state. In stark contrast, both the QN‐HG and QN‐S‐HG groups demonstrated a profound suppression of iNOS expression (3.3% ± 0.2% and 2.9% ± 0.6%, respectively), whereas the blank HG vector sustained elevated levels (13% ± 0.4%; Figure 8E). Reciprocally, CD206 expression was significantly upregulated in the QN‐HG and QN‐S‐HG‐treated wounds, confirming a substantial skewing toward the anti‐inflammatory M2 phenotype (Figure 8F). Collectively, these findings demonstrate that QN‐S‐HG successfully curtails M1 macrophage persistence and actively orchestrates the M1‐to‐M2 phenotypic switch. This potent immunomodulatory shift establishes a favorable microenvironmental niche that facilitates extracellular matrix remodeling and accelerates the overall healing cascade.

## 3 Conclusion

In summary, we have developed a biomimetic nanocomposite hydrogel (QN‐S‐HG) that addresses the multifactorial pathophysiology of diabetic wounds. The neutrophil‐membrane‐coated quercetin nanoparticles confer synergistic anti‐inflammatory, antioxidant, and antibacterial activities with precision targeting to inflamed endothelium and bacterial pathogens, while the Schiff‐base‐grafted SPRC enables on‐demand H_2_S generation in the acidic wound microenvironment to potently stimulate angiogenesis. The hyaluronic acid network, stabilized solely by dynamic hydrogen bonds, provides injectability, self‐healing, and robust tissue adhesion, ensuring conformal wound coverage and sustained local bioavailability. In vivo studies in a diabetic rat full‐thickness wound model demonstrate that QN‐S‐HG significantly accelerates wound closure, promotes M1‐to‐M2 macrophage repolarization, suppresses pro‐inflammatory cytokine expression, enhances neovascularization, and facilitates orderly collagen remodeling with evidence of hair follicle regeneration. By harmonizing targeted pharmacotherapy, gaseous signaling, and adaptive material mechanics within a single biocompatible and easy‐to‐fabricate platform, this work overcomes the limitations of conventional single‐function interventions and establishes a versatile framework for designing microenvironment‐responsive biomaterials. Collectively, QN‐S‐HG represents a clinically promising strategy for diabetic wound care and offers a broadly adaptable paradigm for regenerative medicine, where complex pathological milieus demand multifaceted therapeutic engagement.

## Author Contributions

**Kefan Wu**: Writing – original draft, Methodology, Formal analysis, Data curation. **Xiaoxue Yang**: Methodology, Validation, Formal analysis. **Yonghang Liu**: Methodology, Validation. **Yi Xiao**: Methodology. **Shiyu Meng**: Methodology, Formal analysis. **Ao Li**: Investigation. **Yi Zhun Zhu**: Writing – review & editing, Supervision. **Hui Guo**: Writing – review & editing, Supervision. **Xiaolin Wang**: Writing – review & editing, Supervision, Funding acquisition, Conceptualization.

## Conflicts of Interest

The authors declare no conflicts of interest.

## Supporting information




**Supporting File**: advs78108‐sup‐0001‐SuppMat.docx.

## Data Availability

The data that support the findings of this study are available from the corresponding author upon request.
