# Supplementary material for: Self‐Adaptive Biomimetic Nanocomposite Hydrogel Orchestrates Dual‐Targeting and On‐Demand H2S Gasotransmission for Multifactorial Diabetic Wound Therapy
Source: Adv Sci (Weinh). 2026 Oct 6:e78108. Online ahead of print. doi: 10.1002/advs.78108 (PMC13643095; doi:10.1002/advs.78108)
Supplement: Supplementary file 1 [file ADVS-9999-e78108-s001.docx]

**Supportinng Materials for**

**Self-Adaptive Biomimetic Nanocomposite Hydrogel Orchestrates Dual-Targeting and On-Demand H₂S Gasotransmission for Multifactorial Diabetic Wound Therapy**

Kefan Wu^1^, Xiaoxue Yang^1^, Yonghang Liu, Yi Xiao, Shiyu Meng, Ao Li, Yi Zhun Zhu*, Hui Guo*, and Xiaolin Wang*

School of Pharmacy and State Key Laboratory of Mechanism and Quality of Chinese Medicine, Macau University of Science and Technology, 999078, Macao.

**Corresponding Authors**

**Yi Zhun Zhu**: E-mail: yzzhu@must.edu.mo

**Hui Guo**: E-mail: guohui@must.edu.mo

**Xiaolin Wang**: E-mail: [xilwang@must.edu.mo](mailto:xilwang@must.edu.mo)

1. **Materials**

Poly(lactic-*co*-glycolic acid) (PLGA, lactide: glycolide = 75: 25, molecular weight 75,000 g/mol), Hyaluronic acid (*M*_n_ = 1.5🞨10^6^ g.mol^-1^, dispersity = 2.1, Purity: 98%) were supplied by Macklin Biochemical Technology Co., Ltd. (Shanghai, China), D-(+)-Glucono-1,5-lactone (GdL), sodium hydroxide (NaOH), phenolphthalein, potassium chloride (KCl), urea, ethylene glycol (EG), Sodium Periodate, Dichloromethane, dimethyl sulfoxide (DMSO), Acetone, and quercetin were purchased from Macklin Biochemical Technology Co., Ltd. (Shanghai, China). Anti-iNOS antibody (Catalog No. sc-7271), Anti-ICAM-1 antibody (Catalog No. sc-8439), Anti-GAPDH antibody (Catalog No. sc-365062), and anti-β-Actin antibody (Catalog No. sc 47778) were purchased from Santa Cruz Biotechnology (CA, USA). Recombinant human TNF-α (Catalog No. 570114), recombinant human IL-1β (Catalog No. 579402), and Coomassie Blue Staining Solution (Catalog No. cw0023s) were purchased from Cwbio IT Group (Beijing, China). DIO (3,3′-Dioctadecyloxacarbocyanine perchlorate) (Catalog No. D275) and DiD (1,1′ dioctadecyl-3,3,3′,3′-tetramethylindodicarbocyanine perchlorate) (Catalog No. D307). Human TNF-α ELISA Kit (Catalog No. EK182) and Human IL-1β ELISA Kit (Catalog No. EK101B) were purchased from Multi Sciences (Hangzhou, China). 1-diphenyl-2-picrylhydrazyl (DPPH), Hydrogen Sulfide (H2S) Content Detection Kit were purchased from Solarbio, China. 2',7'-dichlorodihydrofluorescein diacetate (DCFH-DA) and Live/Dead™ BacLight™ Bacterial Viability Kits were purchased from Thermo Fisher Scientific, USA. Washington state probe-1 (WSP-1) was purchased from Cayman Chemical company (Michigan, USA). Resazurin sodium salt was purchased from Sigma-Aldrich, USA. Luria-Bertani (LB) Broth, LB Agar, Dulbecco's modified Eagle medium (DMEM), fetal bovine serum (FBS), horse serum, penicillin-streptomycin stock solutions, and trypsin-EDTA (0.25%) were obtained from Invitrogen, USA. H&E High-Definition Constant Staining Kit, Masson's Trichrome Staining Kit, and primary antibodies to TNF-α, IL-1β, IL-10, and CD31 were purchased from ServiceBio, China. SPRC (purity > 99%) was synthesized according to a previous study ^[1]^.

2. **Neutrophil membrane (NM) derivation**

HL-60 cells were cultured in Iscove’s Modified Dulbecco’s Medium (IMDM) supplemented with 20 % fetal bovine serum (FBS) and 1% penicillin/streptomycin at 37 ◦C with 5% CO_2_. To obtain activated neutrophil-like cells, HL-60 cells were subjected to differentiation using DMSO at a concentration of 1.25 vol% for 4 days. Next, the cells were washed with PBS and centrifuged 3 times at 800 rpm for 10 min. Subsequently, the differentiated HL-60 cells were resuspended in an IB-1 solution composed of 225 mM mannitol, 75 mM sucrose, 30 mM Tris- HCl, 0.5 mM EDTA, and 0.5 % BSA. The differential HL-60 cells were disrupted 150 times using a Dounce homogenizer, followed by centrifugation at 12,000 g for 30 min at 4 ◦C. The supernatant was further centrifuged at 100,000 g for 1 h at 4 ◦C. The BCA protein assay kit was used to quantify the amount of extracted membrane protein. Finally, the collected neutrophil membranes (NM) were stored at 80 ◦C for subsequent analyses.

3. **Synthesis and characterization of NM-NP**

The polymeric cores were prepared with PLGA with the emulsion (o/w)-solvent evaporation method. As previously described, 25 mg of PLGA was dissolved in 1.5 mL dichloromethane (DCM), while 4 mg of Quercetin was dissolved in 0.5 mL acetone, mixed with the PLGA solution, and added dropwise into an aqueous phase (1% w/v PVA). Subsequently, the mixture was sonicated at a power of 130 W for 160 s in an ice bath with vigorous stirring for 3-4 h to allow for organic solvent evaporation. The resulting PLGA NPs were collected by centrifugation (15000 rpm, 30 min) at 4 °C. For the fluorescence experiment, coumarin-6 and DiD were encapsulated into NP at a weight ratio of 1%. For the synthesis of NM-NPs, the HL-60 membrane was mixed with NPs at a membrane-to-core weight ratio of 1:1. The mixture was sonicated in an ice bath at a power of 100 W for 30 s (sonication for 1 s, intervals for 2 s). The product was centrifuged at 15000 rpm at 4 °C for 30 min. Transmission electron microscopy (TEM) was used to observe the morphology of the Q@NPs and Q@NM-NP. The hydrodynamic size and zeta potentials of NPs, NM, and NM-NPs were determined by Zetasizer Nano ZS (Malvern, UK). The surface membrane-associated protein was verified by Coomassie blue staining and Western blot.

4. **Drug loading capacity of NM-NP**

To verify the drug loading capacity of Q@NM-NP, nanoparticles were dissolved in DMSO, and the amount of Quercetin was measured by UV spectrophotometry (Shimadzu UV-2700 spectrophotometer, Japan) at 374 nm. The drug loading (DL) and encapsulation efficiency of the Quercetin-loaded nano formulations were calculated as follows:

$$EE(\%)=\frac{Weight of total Que - Weight of unwrapped Que}{Weight of total Que}\times100\%$$

$$DL(\%)=\frac{Weight of total Que - Weight of free Que}{Weight of nanoparticle}\times100\%$$

5. **Drug release *in vitro***

1 mL of Quercetin, Q@NP, or Q@NM-NP suspensions were placed into dialysis bags (MWCO 12-14 kDa) immersed in 50 mL PBS (pH 7.4) at 37°C and shaken at 100 rpm. At predetermined time points, 2 mL of the solutions were collected, and the same volume of fresh PBS was supplemented. The concentration of Quercetin was determined by UV spectrophotometry, and the cumulative release rate was calculated by the following equation:

where M_t_ stands for the drug released at time t, and M_0_ is the total amount of drug encapsulated in the nanoparticle.

6. **Synthesis of OHA polymer**

OHA polymer was synthesized according to previous work ^[2]^. Briefly, HA (1 g, *M*_n_ = 1.5 🞨 10^6^ g.mol^-1^) was dissolved in deionized water (100 mL). The sodium periodate solution (5 mL, containing 0.5 g) was gradually added dropwise to the HA solution. The reaction was stirred at 25 ◦C in the dark for 6 h. Finally, the excess ethylene glycol (1.2 mL) was introduced to terminate the reaction. After 1 h, the whole system was purified by dialysis membrane (MWCO 3500 Da) against distilled water for 72 h, and the resulting OHA was obtained by lyophilization.

7. **Preparation of the injectable HG hydrogel**

The HG hydrogel was achieved by the physical crosslinking approach. Specifically, 12 wt% HA and 12 wt% OHA were dissolved in a 1 wt% NaOH solution to yield a highly concentrated polymer solution. Then, the GDL was dissolved into the polymer solution to gradually neutralize the pH for hydrogel formation. To prepare the functional composite QN-S-HG hydrogel, 25 mg SPRC was dissolved in the OHA solution for the Schiff base reaction, in which the imine bond was linked between the aldehyde and amino groups. Then, the SPRC-modified OHA and HA were dissolved together in a 1 wt% NaOH solution. Subsequently, the Q@NM-NP were dispersed into the polymer solution, followed by the addition of GDL to neutralize the system and form the QN-S-HG hydrogel.

8. **Mechanical and adhesive properties of the hydrogel**

Compression tests were performed on injectable hydrogel and nanocomposite hydrogel samples using a 50N load cell-equipped electronic universal testing machine (USF-2000A, Shimadzu). Prior to compression testing, the samples were cut into circular shapes with a diameter of 10 mm. The thickness of each sample was measured with a vernier caliper before testing.

For adhesive property evaluation, injectable hydrogels and nanocomposite hydrogels were cut into strips. Adhesion tests were conducted on glass, porcine skin, silicone, copper sheets, and acrylic plates using the same 50 N load cell-equipped electronic universal testing machine (USF-2000A, Shimadzu).

9. **Rheological testing of hydrogel**

Using a rheometer, the rheological behavior of the hydrogel was analyzed under oscillatory temperature mode by cone-plate clamp with a fixed frequency of 10 Hz, a strain of 0.1%, and a temperature of 25°C.

10. ***In vitro* drug release and drug loading capacity of hydrogel**

1 g of QN-HG was placed into dialysis bags (Molecular weight cut-off: 12-14 kDa) immersed in 50 mL PBS (pH 7.4) at 37 °C and shaken at 100 rpm. At predetermined time points, 2 mL of the solutions were collected, and the same volume of fresh PBS was supplemented. The concentration of Quercetin was determined by UV spectrophotometry,

The S-HG hydrogel was placed into dialysis bags (MWCO 12-14kDa) immersed in 50 mL PBS (pH 7.4 and 5.5) at 37°C and shaken at 100 rpm. At predetermined time points, 2 mL of the solutions were collected, and the same volume of fresh PBS (pH 7.4 and 5.5) was supplemented. To detect the concentration of SPRC, the sample solution was analyzed by ultra-performance liquid chromatography (UPLC, Agilent’s 1290 In finity Quat UHPLC System). The Ultimate AQ-C18 HPLC column (5 μ m, 4.6 × 250 mm) was used with column temperature set at 25 ◦ C. The mobile phase includes acetonitrile (A) and buffer (B) containing 10 mM sodium 1-heptane sulfonate and 20 mM monopotassium phosphate (pH =2.4). The injection volume of each sample was 10 μ L, and the flow rate of the current mobile phase (A: B = 1: 9) was set at 1 mL/min. The concentration of SPRC was detected at a wavelength of 210 nm., and the cumulative release rate was calculated by the following equation:

where M_t_ stands for the drug released at time t, and M_1_ is the total amount of drug encapsulated in the hydrogel.

The drug loading (DL) and encapsulation efficiency of the S-HG hydrogel were calculated as follows:

$$EE(\%)=\frac{Weight of total SPRC - Weight of unwrapped SPRC}{Weight of total SPRC}\times100\%$$

$$DL(\%)=\frac{Weight of total SPRC - Weight of free SPRC}{Weight of hydrogel}\times100\%$$

11. **Intracellular H2S detection**

Washington state probe-1 (WSP-1) was used to trace H2S *in vitro*^[3]^. HUVEC cells were seeded into a 6-well plate overnight followed by pretreatment under LPS-stimulated for 12 h). After co-incubating with HG, S-HG, QN-HG, and QN-S-HG for 4 h, cells were washed with PBS. Afterward, cells were treated with WSP-1 (50 μМ) at 37 ◦C for 1 h in the dark. The fluorescent signals of WSP-1 were observed by a fluorescent microscope. Moreover, the Hydrogen Sulfide (H_2_S) Content Detection Kit (Solarbio, China) was also used to measure H_2_S *in vitro*.

12. **Cytotoxicity and hemolysis assay**

NIH 3T3 and HUVEC cells were cultured in 24-well plates at a density of 2 × 10^^5^ and incubated with HG, S-HG, QN-HG, and QN-S-HG hydrogels at 37°C for 24 h. Then, the medium was replaced with Alamar Blue solution diluted 100-fold in DMEM and incubated for another 4 h at 37°C. The absorbance of each well was measured at 570 nm and 600 nm by a microplate reader (SpectraMax ID5, Molecule Devices, USA). The cell viability rate (V_c_) was calculated according to the equation as follows:

Where As and Ac denote the absorbance of the sample and control group, respectively.

To investigate hemocompatibility, red blood cells (RBC) were isolated from fresh rat whole blood by centrifuging at 3000 rpm for 5 min, rinsed, and resuspended in 2% RBC suspension in PBS (10 mM, pH = 7.4). Subsequently, HG, S-HG, QN-HG, and QN-S-HG hydrogels were dropped into 2% RBC suspensions. Meanwhile, PBS and distilled water were accepted as the negative and positive controls. After incubation at 37 °C for 3 h and centrifugation at 1500 rpm for 10 min, the absorbance of the supernatant of each group was assayed by a microplate reader at 540 nm, and the hemolysis rate was calculated as follows:

Where A_sample_, A_negative_, and A_positive_ denote the absorbances of the sample, negative control, and positive control group, respectively.

13. **Antioxidant effects *in vitro***

The antioxidant effects were tested by 1, 1-diphenyl-2-picrylhydrazyl (DPPH). Briefly, different treatment groups were added to 2 mL of DPPH solution. The mixture was shaken and kept for 40 min at room temperature in the dark. The absorbance of the mixture was measured by a microplate reader at 517 nm (SpectraMax ID5, Molecule Devices, USA). The DPPH radical scavenging ability was calculated using the following formula:

Where A_0_ and A_1_ denote the absorbances of the control and sample group, respectively.

The intracellular ROS scavenging capacity of OHG hydrogels was tested on RAW 264.7. The cells were cultured in a 24-well plate for 24 h. Then HG, S-HG, QN-HG, and QN-S-HG were co-incubated for 6 h. After washing with PBS, cells were cultured with DCFH-DA for 30 min. The fluorescence in cells was photographed using an inverted fluorescence microscope. The fluorescence intensity was measured by ImageJ.

14. **Antibacterial performance *in vitro***

The antibacterial activities of the hydrogel were tested against Staphylococcus aureus (*S.aureus*) and Escherichia coli (*E.coli*). Briefly, a certain amount of hydrogel precursor solution was co-incubated with bacterial suspension (10μl, 10^6^ CFU/ml) for 6 h. The group of bacteria without treatment was set as the control. The number of colonies on the plate was counted with ImageJ software. The sterilizing rate (S_b1_ and S_b2_) was calculated by the following formula:

Where Ab is the absorbance of the LB broth, Ac and As represent the absorbance of the control and sample groups, respectively.

Where Nc and Ns represent the number of colonies from the control and sample group, respectively.

To evaluate the disruption of bacterial biofilm, 200 μL of bacterial solution was pipetted into a 96-well plate. Different gels were added according to grouping and incubated in a constant temperature incubator at 37 °C for 24 h. At the end of the incubation, the LB broth was removed and the biofilm was gently rinsed with PBS. Next, biofilm production was quantitatively evaluated by crystal violet staining. 200 μL 50% crystal violet was used for biofilm staining for 10 min, followed by adequate drying of the bacterial biofilm, and finally the crystal violet was dissolved in 75% ethanol and the absorbance at 595 nm was measured.

Additionally, the four groups were staibed with SYTO-9 and PI for 30 min at room temperature and photographed via CLSM to obtain three-dimensional fluorescence images.

15. **Macrophage polarization *in vitro***

RAW 264.7 cells were activated with 1 μg mL^-1^ LPS for 2 h to induce inflammation. Next, hydrogels containing equal amounts of Quercetin were added to the wells and incubated for another 4 h. The macrophages were further incubated for another 18 h. Then, the cells were collected by cell scraping using RIPA lysis buffer containing a protease inhibitor cocktail. The PVDF membranes were incubated with the corresponding primary antibodies against iNOS and Arg-1 and then incubated with relevant secondary antibodies. Protein bands were analyzed by ImageJ software.

16. **Cell migration ability *in vitro***

HaCaT cells were seeded in a 24-well plate to form a confluent monolayer. The cells were scratched with a sterilized 200 μl pipette tip to form a wound and washed with PBS to remove floating cell debris. The cells were cultured with HG, S-HG, QN-HG, and QN-S-HG in DMEM supplemented with 1% FBS. Representative photographs were captured, and the wound areas were calculated using ImageJ at times of 12 h, 24 h, and 36 h. The cell migration rate (M_c_) was calculated according to the following equations:

Where a_0_ and a_t_ denote the scratch area at the initial time and at specific times, respectively.

17. **Ethics approval and consent to participate**

The experimental protocol was established according to the ethical guidelines of the Helsinki Declaration and was approved by the Ethics Committee of the Animal Center of Macau University of Science and Technology (MUST-GDST-20240718002). Written informed consent was obtained from individual or guardian participants.

18. **Angiogenic effect *in vitro***

The *in vitro* angiogenic ability of HUVECs was verified through a tube formation assay using Matrigel (10 mg/mL, Corning Inc, Corning, NY, USA). HUVECs were cultured in 96-well plates at a density of 2$\times$10^4^. The cells were starved in DMEM without serum for 24 h before the assay. Afterward, each cell was coated with 100 μl of Matrigel, followed by different treatment groups for 6 h. Representative photos of the tube structure were taken by an inverted microscope, and the branch points of blood vessels were measured by ImageJ software.

19. ***In vivo* diabetic wound experiment**

The male SD (Sprague Dawley) rats (180-200 g) were intraperitoneally injected with streptozotocin (STZ) (65 mg/kg) after an overnight fast. Blood glucose was measured using a glucose meter on days 3,7,10,14. The mice were considered diabetic when non-fasted blood glucose levels consistently exceeded 16.7 mM. The diabetic rats were anesthetized and their hair was shaved. A full-thickness cutaneous wound with a diameter of 9 mm was created on the Doral area of each rat, according to previous research. Wounds were treated using different types of HG hydrogels (S-HG, QN-HG, QN-S-HG). The steel ring, which was sutured in the dorsal area of each rat, was used to avoid the automatic contraction of epidermal tissue. At predetermined time points, the wounds were captured with a digital camera, and the wound area was counted in terms of ImageJ software. The wound-healing rate was evaluated by the wound closure rate (CR), which was calculated using the following formula:

Where A_0_ and A_t_ represent the original wound area and the wound area at a certain time interval, respectively.

The skin tissue surrounding the wound edges was collected and analyzed by H&E staining and Masson’s trichrome staining to further evaluate the improvement of tumor necrosis factor-α (TNF-α), Interleukin-1β (IL-1β) and Interleukin-10 (IL-10). Platelet endothelial cell adhesion molecule-1 (CD31) staining was performed to evaluate the angiogenesis of wound healing.

20. **Statistical analysis**

Statistical analysis of data from the experiments was carried out using SPSS statistical software (IBM, USA) and one-way ANOVA. All data were presented as mean ± standard deviation (Mean ± SD). Experimental data were regarded as significantly different and were statistically significant when P < 0.05 (*), P < 0.01 (**) and P < 0.001 (***) in the results. All data can be consulted in the main text or the supplementary information.

**Figure S1.** Encapsulation efficiency and drug loading of Q@NM-NP. Data were expressed as means ± SD, n =3.

**Figure S2.** **Cellular internalization of Q@NM-NP.** A) ICAM-1 protein expression and B) corresponding quantification by ImageJ. Data were expressed as means ± SD, n =3. Statistical significance among various groups was determined by one-way ANOVA with LSD test (**p < 0.01).

**Figure S3. Cellular uptake of Q@NM-NP in HUVEC cells.** A) Cellular uptake of Q@NP and Q@NM-NP in HUVEC cells treated with TNF-α and B) Corresponding mean fluorescence intensity (MFI) analyzed by ImageJ. Scale bar = 25 μm. Data were expressed as means ± SD, n =3. Statistical significance among various groups was determined by one-way ANOVA with LSD test (**p < 0.01).

**Figure S4. The therapeutic potential of Q@NM-NP *in vitro*.** A) Binding kinetics of (A) TNF-α, (B) IL-1β. Data were expressed as means ± SD, n =3.

**Figure S5. Characterization of OHA.** A) The Fourier transform infrared spectra of HA and OHA at 4000-600 cm^-1^. B) The ^1^H NMR of OHA.

**Figure S6.** The ^1^H NMR of S-HG in D_2_O.

**Figure S7.** Encapsulation efficiency and drug loading of S-HG. Data were expressed as means ± SD, n =3.

**Figure S8.** The quercetin release kinetics from QN-S-HG gel. Data were expressed as means ± SD, n =3.

**Figure S9.** The H_2_S generation-time curve in inflammatory HUVEC cells for a period time treated with HG, S-HG, QN-HG, and QN-S-HG, respectively. Data were expressed as means ± SD, n =3.

**Figure S10.** H_2_S production in inflammatory HUVEC cells with HG, S-HG, QN-HG, and QN-S-HG (blue: nucleus; green: WSP-1 as H_2_S probe; scale bar = 50 μm).

**Figure S11.** Viscoelastic behavior and gelation time of QN-S-HG alkaline solutions upon adding GdL (GdL/NaOH = 1/1, molar ratio; frequency: 1 Hz; strain: 15%).

**Figure S12. The cell viability of Q@NM-NP.** A) The NIH3T3 cells co-incubated with different concentrations of Q@NM-MP. B) The cell viability of HUVEC cells co-cultured with different concentrations of Q@NM-MP. Data were expressed as means ± SD, n =3.

**Figure S13.** Hemolysis rates of Q@NM-MP. Data were expressed as means ± SD, n =3.

**Figure S14. Antibacterial performance of Q@NM-MP.** A) Representative images of bacterial colonies on agar plates. B) Corresponding bacterial viability of different NPs against *S.aureus* and C) *E.coli*. Scale bar = 20 mm. Data were expressed as means ± SD, n =3. Statistical significance among various groups was determined by one-way ANOVA with LSD test (ns: non-significant, **p < 0.01; ***p < 0.001).

**Figure S15. Cell migration of QN-S-HG gel in HUVEC cells.** Quantitative statistics of HUVEC migration at A) all time intervals and B) after 12 h. Scale bar, 300 μm. C) Representative images of HUVEC cell migration treated with HG, S-HG, QN-HG, and QN-S-HG. Control (+) group: LPS stimulation. Control (-) group: no LPS stimulation. Data were expressed as means ± SD, n =3. Statistical significance among various groups was determined by one-way ANOVA with LSD test (***p < 0.001).

**Figure S16. DPPH scavenging efficiency of Q@NM-NP.** A) Representative photos of DPPH scavenging. B) Quantitative statistics on DPPH scavenging. Data were expressed as means ± SD, n =3.

**Figure S17.** The weight and blood glucose of diabetic wound rats.

**Figure S18. H&E and Masson staining analysis.** A, B) Panoramic view of H&E and Masson staining of wounds after different treatments on day 7. Scale bar = 50 μm. Data were expressed as means ± SD, n =6.

**Figure S19.** Representative images of immunofluorescence staining of iNOS (Green) and CD206 (Red) on days 7 and 14. Scale bar = 100 μm.

**References:**

[1]	Q. Wang, H.-R. Liu, Q. Mu, P. Rose, Y. Zhu, *J. Cardiovasc. Pharm.* **2009**, *54*, 139.

[2]	L. Rong, Y. Liu, Y. Fan, J. Xiao, Y. Su, L. Lu, S. Peng, W. Yuan, M. Zhan, *Carbohydr. Polym.* **2023**, *310*, 120721.

[3]	X. Zhang, H. Yang, Y. He, D. Zhang, G. Lu, M. Ren, Y. Lyu, Z. Yuan, S. He, *ACS Nano* **2025**, *19*, 7350.
